# Extraction of lotus fibres from lotus stems under microwave irradiation

**DOI:** 10.1098/rsos.170747

**Published:** 2017-09-06

**Authors:** Cheng Cheng, Ronghui Guo, Jianwu Lan, Shouxiang Jiang

**Affiliations:** 1 College of Light Industry, Textile and Food Engineering, Sichuan University, No. 24 South Section 1, Yihuan Road, Chengdu, China; 2 Institute of Textiles and Clothing, The Hong Kong Polytechnic University, Hung Hom, Kowloon, Hong Kong, China

**Keywords:** lotus fibres, microwave, component, hydrogen peroxide, moisture regain

## Abstract

An efficient technology for preparing lotus fibres under microwave irradiation was developed. The lotus fibres were characterized by scanning electron microscopy, Fourier transform infrared spectrometry, X-ray diffraction and thermogravimetry. Lotus fibres prepared are a kind of hollow fibres which are composed of a superfine fibre and an external shell. The effect of the treatment time with hydrogen peroxide under microwave irradiation on components, whiteness, moisture regain, removal rate of impurities, fineness, tensile strength and breaking elongation of lotus fibres was investigated. The results show that the cellulose content in lotus fibres increases with increase in treatment time. Whiteness and moisture regain of lotus fibres increase with increase in treatment time with hydrogen peroxide. The removal rate of impurities and the fineness of lotus fibres are improved after they are treated with hydrogen peroxide. Microwave irradiation is supposed to be an efficient method for producing lotus fibres.

## 1. Introduction

Nowadays, the demands for environment-friendly and fully biodegradable sustainable materials have substantially increased in various industries with the enhancement of people's consciousness of global environmental and energy problems. It has become a significant topic to exploit and study a new natural resource which is green and environment-friendly. Lotus fibre is a kind of natural fibre which is usually extracted from the lotus stem and the lotus root. Currently, a considerable amount of lotus stems are left in the pond to be wasted after the blossom season or the harvest of lotus roots. These residues could provide abundant natural cellulose resources which can be used in the textile, paper, medical and construction industries. Nowadays, lotus fibres are being used to manufacture luxury garments. Garments produced from lotus fibres are becoming more and more popular because they are environment-friendly and comfortable. In addition, lotus fibres have also been widely used as porous and composite materials [1,2].

Currently, extraction by hand is the main method for preparing lotus fibres [3]. However, the low efficiency of manual preparation limits large-scale use of lotus fibres. Developing convenient and efficient methods for preparing lotus fibres has attracted the attention of researchers more and more. Extraction with sodium hydroxide (NaOH) is considered one of the most widely useful and cost-effective methods for preparing natural fibres. Various natural plant fibres such as cotton, hemp, alfa, flax and ramie are obtained with sodium hydroxide [4–7]. Sodium hydroxide is also used for degumming of lotus fibres. However, lotus fibres extracted with sodium hydroxide contain too much of non-cellulose substances [8]. To obtain natural fibres rich in cellulose, chemical reagents such as hydrogen peroxide (H_2_O_2_), sodium hypochlorite (NaClO), sodium chlorite (NaClO_2_), sodium perchlorate and benzoate have been widely used for degumming and purification of natural fibres in the textile industry [9]. Compared with other chemical reagents, hydrogen peroxide is more popular for the degumming of fibres because it is a kind of chlorine-free bleaching agent and only produces carbon dioxide and water, which are environment-friendly.

The microwave irradiation method is very popular for its high speed and convenience, when compared with conventional heating [10]. Currently, microwave irradiation is being widely applied in the food, textile and chemical industries because of its ease of operation and low cost. Microwave technology is being used to extract fibres from plants to improve efficiency in fibre preparation [11]. Preparing fibres with hydrogen peroxide under microwave irradiation has become more and more popular because it is an environment-friendly and efficient method for obtaining cellulose resources [12,13]. However, to the best of our knowledge, there are no reports on preparing lotus fibres from lotus stems with hydrogen peroxide under microwave irradiation.

In this study, lotus fibres were obtained from lotus stems after the latter were treated with sodium hydroxide followed by hydrogen peroxide under microwave irradiation. Morphology, chemical structure, crystal structure and thermal properties of lotus fibres were characterized using scanning electron microscopy (SEM), Fourier transform infrared (FTIR) spectroscopy, X-ray diffraction (XRD) and thermogravimetry (TG), respectively. In addition, the influence of treatment time on components, whiteness, moisture regain, removal rate of impurities, fineness, tensile strength and breaking elongation of lotus fibres was investigated.

## 2. Experimental

### 2.1. Material

Sodium hydroxide and hydrogen peroxide with a grade of analytical purity were purchased from Kelong Reagent Co. Ltd. Lotus stems were collected from agricultural waste after harvesting from Meishan City, China.

### 2.2. Preparation of lotus fibres under microwave irradiation

Fresh lotus stems were washed and dried at room temperature. Dried lotus stems were cut into 5 cm lengths, and 2 g samples were placed into 100 ml of NaOH solution (20 g l^−1^) and treated under microwave irradiation for 20 min. The frequency and power of the microwave generator were 2450 MHz and 750 W, respectively. The lotus stems were then washed in deionized water to neutral pH. After that, the samples were separated from the lotus stems after the latter were squeezed and rinsed. Finally, the samples were dried at 50°C and named as raw lotus fibres.

Raw lotus fibres (2 g) were treated in 100 ml H_2_O_2_ (15 wt%) under microwave irradiation for different time durations. Subsequently, the samples were squeezed and rinsed to remove debris of epidermis and other impurities. Finally, the lotus fibres were rinsed to neutral pH and dried at 50°C.

### 2.3. Characterization

The removal rate of impurities of the samples was calculated according to the following equation:

2.1 $$W=\frac{M_{0}-M_{1}}{M_{0}}\times 100\%,$$

where *W* is the removal rate of impurities, *M*_0_ is the initial mass (g) of lotus stems and *M*_1_ is the mass (g) of lotus fibres after being treated with sodium hydroxide for a period of time.

The chemical structure of the lotus fibres was investigated with a Nicolet 6700 FTIR spectrophotometer for each measurement over the spectral range of 500–4000 cm^−1^ with a resolution of 4 cm^−1^. The morphology of the lotus fibres was observed by SEM (JSM-5900LV). TG was carried out under nitrogen flow at a heating rate of 10°C min^−1^ from 30°C to 750°C (DTG-60, Shimadzu, Japan). The crystal structure of the fibres was determined by XRD (X’ Pert Pro MPO) using CuKα radiation at 40 kV and 50 mA (*λ* = 1.54 Å). The diffraction angle (2*θ*) scanned was changed from 4° to 50°. The ratio of crystalline area to total diffracted area was taken as the crystallinity. The crystallinity index (CI) was calculated according to the following equation [14]:

2.2 $$\text{CI}=\frac{I_{c}-I_{\mathrm{am}}}{M_{c}}\times 100\%,$$

where *I*_c_ and *I*_am_ represent the readings at an angle of 2*θ* to the crystalline and the amorphous region, respectively.

Components of the lotus fibres were investigated according to the standard GB 5889-86. Lignin in the lotus fibres was determined according to the standard ASTM D1106-96. Whiteness of the lotus fibres was measured by a digital display white meter (SBDY1).

Moisture regain of the lotus fibres was determined according to the standard ASTM D2495. The samples were dried at 105 ± 2°C for 1 h and then weighed. The samples were then conditioned in the room with 65% relative humidity at 20°C. The samples were weighed per 5 min until the change of weight was less than 0.1%. The moisture regain was calculated according to the following equation:

2.3 $$M_{a}\;(\%)=\frac{W_{\mathrm{aw}}-W_{d}}{W_{d}}\times 100,$$

where *M*_a_ is the moisture regain of lotus fibres, *W*_aw_ is weight after absorbing water and *W*_d_ is the weight of the samples after drying.

Fineness of the fibres was measured according to the standard ASTM D2252-05. Tensile strength and breaking elongation of the lotus fibres were evaluated according to the standard ASTM D3822. Gauge length and loading speed were 1 cm and 3 mm min^−1^, respectively. Tests were repeated 50 times for each sample and the average value was calculated as the result.

## 3. Results and discussion

### 3.1. Removal rate of impurities

**Figure 1.**
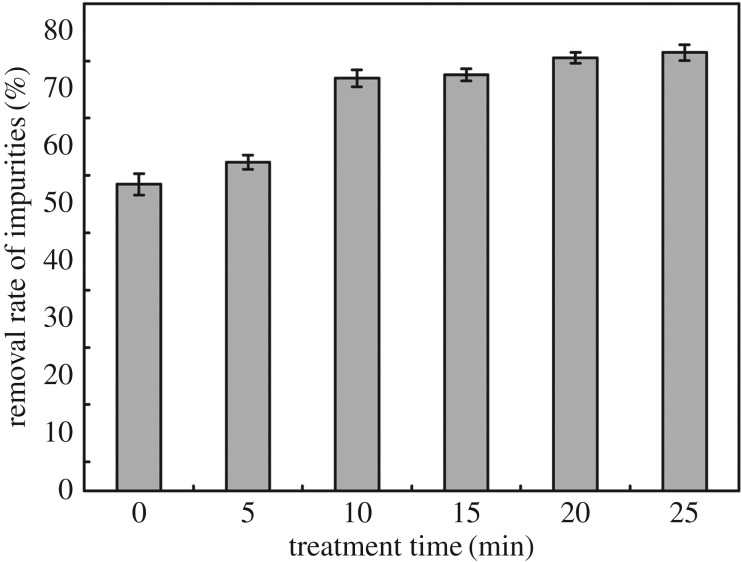
Influence of treatment time on removal rate of impurities of lotus fibres.

The influence of treatment time with hydrogen peroxide under microwave irradiation on the removal rate of impurities of lotus fibres is shown in figure 1. The removal rate of impurities increases rapidly from 53% to 73% when the treatment time is increased from 0** **min to 10 min, and then reaches 77% when raw lotus fibres are treated for 25 min. Lotus fibres are distributed inside the lotus stem and covered with waxes, pectin and some lignin. These non-cellulose impurities such as pectin and water-soluble substances are the main ingredients of walled ground parenchyma cells in the lotus stem [15]. Most of the pectin, waxes and water-soluble substances are removed with the dissolution of walled ground parenchyma cells in the lotus stem during preparation of lotus fibres. In addition, dissolution of hemicellulose and lignin in hydrogen peroxide can also improve the removal rate of impurities.

### 3.2. Components

**Figure 2.**
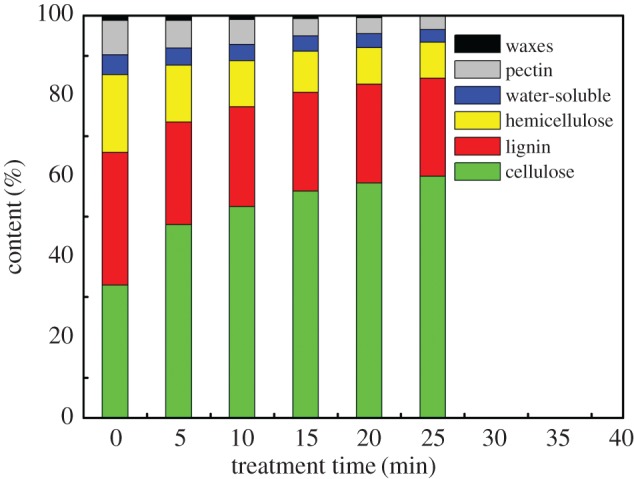
Components of lotus fibres treated with hydrogen peroxide for different time durations.

Figure 2 shows variation of components in lotus fibres prepared with hydrogen peroxide for different time durations. Cellulose and non-cellulose contents in lotus fibres increase and decrease with increase in treatment time, respectively. This can be a result of the generation of hydroxyl radicals from hydrogen peroxide under microwave irradiation. Decomposition reactions of hydrogen peroxide under microwave irradiation are shown in the following equations [16]:

3.1 $${\text{H}}_{2}{\text{O}}_{2}\overset{\mathrm{microwave}}{\to }2{\mathrm{HO}}^{\cdot }$$

3.2 $${\text{H}}_{2}{\text{O}}_{2}+{\text{HO}}^{\cdot }\to {\text{HOO}}^{\cdot }+{\text{H}}_{2}\text{O}$$

3.3 $$\text{and}\;\;{\text{HO}}^{\cdot }+{\text{HOO}}^{\cdot }\to {\text{O}}_{2}+{\text{H}}_{2}\text{O}$$

The contents of waxes and water-soluble substances of lotus fibres treated with hydrogen peroxide for 25 min are increased to 0.03% and 3.48%, respectively. The pectin content in lotus fibres before being treated with hydrogen peroxide is 8.51%. However, the pectin content is only 3.49% when the sample has been treated with hydrogen peroxide for 25 min. Waxes, water-soluble substances and pectin are removed due to the disruption of chemical bonds among the molecules of these substances and the contents of these substances significantly decrease.

Hemicellulose in lotus fibres decreases rapidly from 19.29% to 9.12% with increase in treatment time from 0 min to 25 min. The phenomenon can be explained by the fact that hemicellulose, which is a heteropolymer of xylose, l-arabinose and galactose, can be dissolved in a solution of hydrogen peroxide. Lignin decreases from 32% to 25% with increase in treatment time from 0 min to 5 min, which is due to the removal of lotus stem epidermis containing lignin. Lotus stem epidermis is damaged into debris, and then is completely damaged during rinsing in deionized water when the time of treatment reaches 5 min. However, lignin in lotus fibres is hardly removed due to its complicated chemical structure. In addition, combination between lignin and cellulose in lotus fibres is too strong to be broken by the hydrogen radical. Therefore, the lignin content in lotus fibres remains almost constant with further increase in treatment time [17]. The cellulose content in lotus fibres increases from 33.01% to 60.92% due to the removal of non-cellulose impurities. The results indicate that purification of raw lotus fibres can efficiently enhance the cellulose content in lotus fibres.

### 3.3. Surface morphology

**Figure 3.**
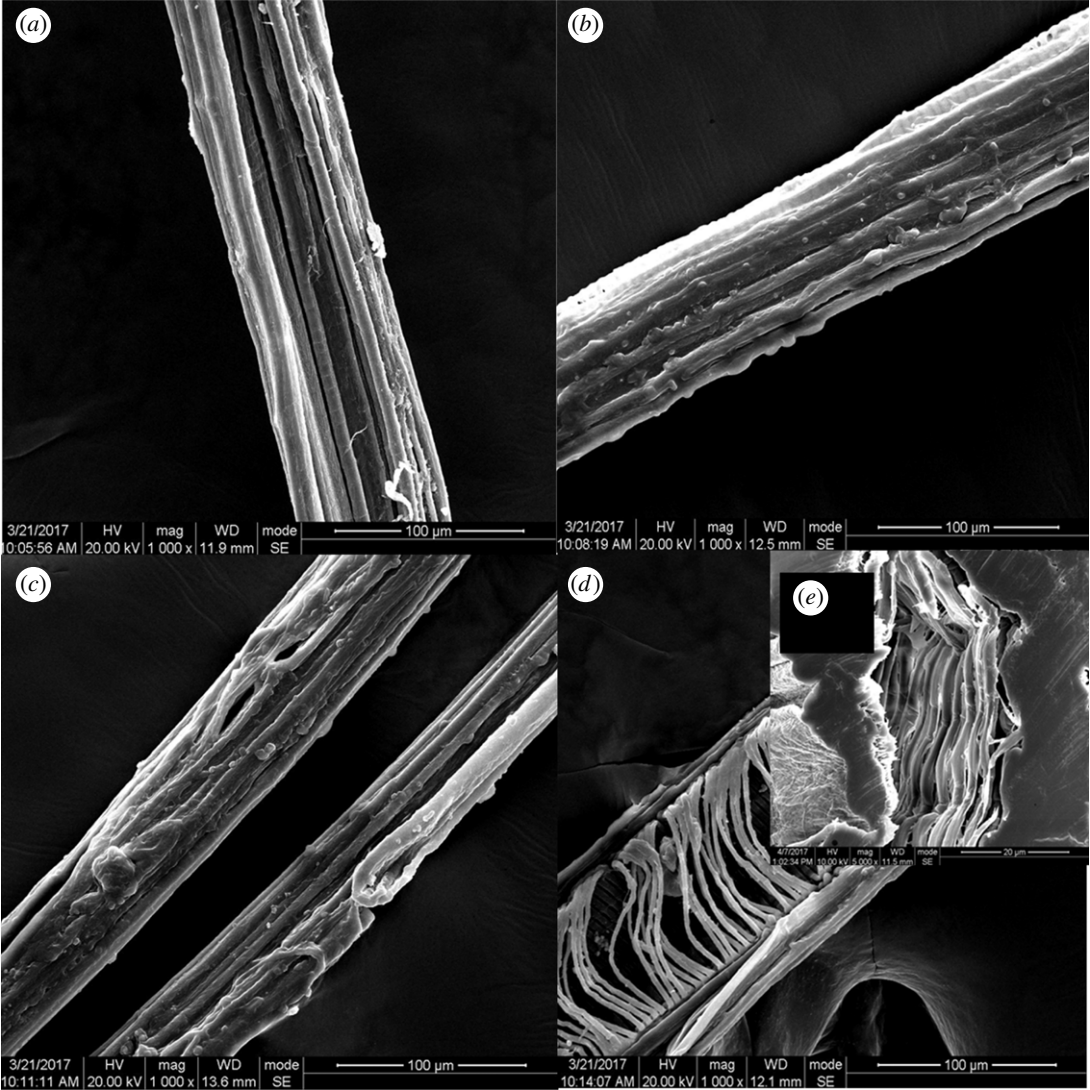
Morphology of lotus fibres: (*a*) raw lotus fibres; lotus fibres after being treated with hydrogen peroxide for (*b*) 5 min, (*c*) 15 min and (*d*) 25 min; and (*e*) cross-sectional morphology of lotus fibres.

The morphologies of raw lotus fibres and lotus fibres after being treated with hydrogen peroxide for 5 min, 15 min and 25 min are shown in figure 3. The surface of raw lotus fibres is smooth with many longitudinal grooves. The diameter of raw lotus fibres ranges from 75 µm to 80 µm. Impurities can be observed on the surface of raw lotus fibres (figure 3*a*). The surface of lotus fibres after being treated with hydrogen peroxide for 5 min and 15 min is rough with numerous protuberances. The diameter of lotus fibres after being treated for 5 min changes from 68 µm to 75 µm, whereas after being treated for 15 min, it changes from 45 µm to 55 µm. Impurities on the surface of lotus fibres are fewer than those on raw lotus fibres. In addition, the depth of grooves on the surface of lotus fibres is shallower than that on raw lotus fibres (figure 3*b*,*c*). Transversely distributed superfine fibres with diameters ranging from 2 µm to 4 µm are observed from the inside of the lotus fibres after the external shell of the lotus fibres is damaged when the treatment time is 25 min (figure 3*d*).

The cross section of lotus fibres is shown in figure 3*e*. The cross-sectional shape of lotus fibres is irregular and the edge of lotus fibres is full of wrinkles. Lotus fibres are hollow and composed of an external shell and an inner superfine fibre. The thickness of the external shell is 8 µm to 20 µm. Superfine fibres are densely distributed on the interior walls of the shell in a spiral manner**.**

**Figure 4.**
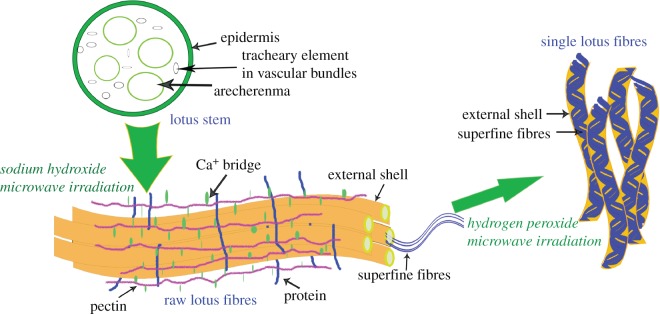
Preparation process of lotus fibres under microwave irradiation.

The preparation process of lotus fibres under microwave irradiation and the morphological character of lotus fibres are shown in figure 4. Lotus fibres are abundant in the stems of the leaf and flower and in the root of the lotus plant [18]. Raw lotus fibres are separated from lotus stems after being treated with sodium hydroxide under microwave irradiation. Raw lotus fibres contain waxes, pectin and other non-cellulose substances. Therefore, raw lotus fibres are fibre bundles connected by non-cellulose substances. Lotus fibres are a kind of hollow fibre, which is composed of internal superfine fibres and an external shell. Waxes, pectin and other non-cellulose substances are removed due to the breaking of hydrogen bonds between these non-cellulose substances and cellulose, and the fibre bundles are divided into single fibres after being treated with hydrogen peroxide. Lotus fibres rich in cellulose are obtained after being treated with sodium hydroxide followed by hydrogen peroxide under microwave irradiation.

### 3.4. X-ray diffraction analysis

**Figure 5.**
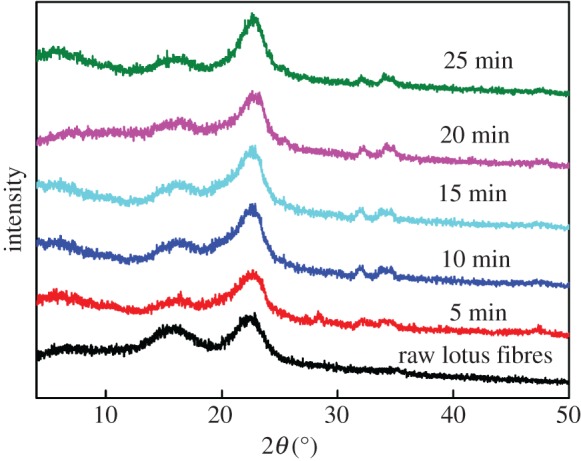
XRD patterns of lotus fibres treated with hydrogen peroxide for different time durations.

XRD patterns of lotus fibres prepared by different treatment time durations are shown in figure 5. A sharp peak at 22° and a wide peak between 14° and 17° are observed from all the spectra of lotus fibres. The result shows that lotus fibres belong to the cellulose I structure [19]. However, variations in peak intensity indicate that crystallinity of lotus fibres changes as they are treated with hydrogen peroxide under microwave irradiation. The peaks at 16°, 32° and 34° are attributed to a secondary peak for the amorphous region of cellulose, whereas the prominent peak at 22° represents the crystalline region of cellulose [20].

Crystallinity and the CI of lotus fibres are improved after they are treated with hydrogen peroxide as shown in table 1. The crystallinity of lotus fibres increases from 40.94% to 62.60%. In addition, the CI of lotus fibres increases from 46.40 to 60.64. The result indicates that the ratio of the amorphous region in lotus fibres decreases with increase in treatment time. The phenomenon can be explained by the fact that the amorphous region of cellulose in lotus fibres is dissolved during their treatment with hydrogen peroxide and their crystalline structure is rearranged and the crystallinity of cellulose increases as the treatment time is prolonged [21].

**Table 1.** Crystallinity and CI of lotus fibres treated for different time durations.

treatment time	untreated	5 min	10 min	15 min	20 min	25 min
crystallinity (%)	40.94	55.24	58.29	60.29	62.86	62.60
CI (%)	46.40	56.38	58.39	59.21	58.38	60.64

### 3.5. Fourier transform infrared spectrometry analysis

**Figure 6.**
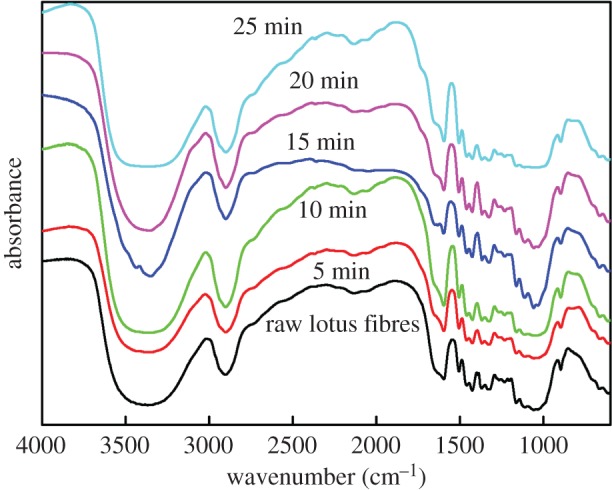
FTIR spectra of lotus fibres treated with hydrogen peroxide for different time durations.

FTIR spectra of lotus fibres treated with hydrogen peroxide for different time durations are illustrated in figure 6. Broad characteristic peaks at 3370–3390 cm^−1^ belong to the stretching of OH– groups of cellulose I, whereas characteristic peaks at 2890–2910 cm^−1^ correspond to CH– and CH_2_– asymmetric stretching vibrations [22]. Characteristic peaks of all the samples at 1020–1040 cm^−1^ are attributed to C–O–C stretching vibration of the pyranose ring and glycosidic ether linkages between glucose units in cellulose, respectively. The peaks appearing at 890–900 cm^−1^ can be ascribed to β glycoside bonds of cellulose [23]. In addition, absorption peaks at 1650–1660 cm^−1^ and 1590–1600 cm^−1^ are assigned to oxygenous groups and benzene skeleton vibration in lignin, respectively. The peak at 1150–1170 cm^−1^ is ascribed to C─O symmetric bridge stretching of uronic ester groups in hemicelluloses [24].

### 3.6. Thermal analysis

**Figure 7.**
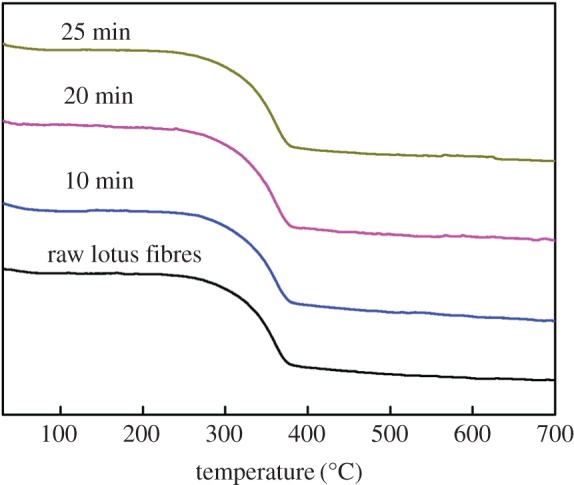
TG curves of lotus fibres treated with hydrogen peroxide for different time durations.

The thermal properties of lotus fibres treated with hydrogen peroxide for different times are illustrated in figure 7. The initial weight loss stage of lotus fibres at 100°C to 110°C results from volatilization of water and surface oil. The second stage of weight loss is observed between 360°C and 380°C due to thermal depolymerization of hemicellulose and pectin, and cleavage of glycosidic linkages of cellulose. The third stage of weight loss, which occurs at 380°C to 650°C, may result from the breaking down of main chain groups of lotus cellulose and the slow decomposition of the high molecular weight complex of lignin [25]. Lotus fibres after being treated with hydrogen peroxide are almost decomposed when the temperature is elevated to 700°C; however, raw lotus fibres remain more residual than lotus fibres. This phenomenon can be explained by the fact that lignin and hemicellulose contents in raw lotus fibres are much more than that in lotus fibres after being treated with hydrogen peroxide.

### 3.7. Whiteness

**Figure 8.**
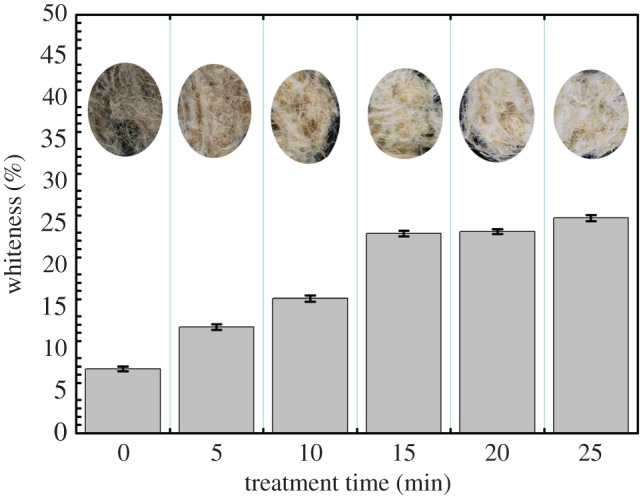
Influence of the treatment time with hydrogen peroxide on the whiteness of lotus fibres.

Whiteness of lotus fibres is shown in figure 8. Raw lotus fibres are brown and their whiteness is 7.7%. The colour of lotus fibres changes to canary, and the whiteness of lotus fibres significantly increases with increase in treatment time due to the bleaching effect of hydroxyl radicals generated from hydrogen peroxide on lotus fibres. The concentration of hydroxyl radicals increases with the decomposition of hydrogen peroxide and then reaches a maximum. Hydrogen radicals from hydrogen peroxide can oxidize the carbonyl group or quinoid in the side chains of lignin; consequently, the chromophore structure in lignin is damaged and the whiteness of the lotus fibre is improved [26,27].

### 3.8. Moisture regain

**Figure 9.**
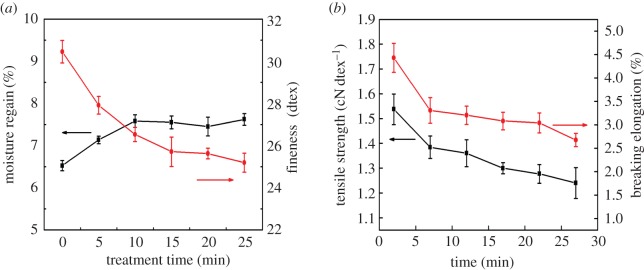
Influence of the treatment time with hydrogen peroxide on the properties of lotus fibres: (*a*) moisture regain and fineness; (*b*) tensile strength and breaking elongation.

The influence of treatment time with hydrogen peroxide on moisture regain of lotus fibres is illustrated in figure 9*a*. The moisture regain of lotus fibres is 7.62%, which is close to that of cotton (7.48%) but lower than that of flax (8.51%) [28]. Lotus fibres possess excellent hydrophilic ability due to abundant –OH in macromolecular chains of cellulose in them. In addition, numerous grooves and interspaces on the surface of lotus fibres can also improve their hydrophilic ability. The moisture regain of lotus fibres increases rapidly and then remains constant with increase in treatment time. The phenomenon can be explained by the fact that interspaces and surface area in lotus fibres increase with increase in treatment time to 15 min, and then reach a maximum when the treatment time is 15 min.

### 3.9. Fineness

The influence of treatment time with hydrogen peroxide on the fineness of lotus fibres is shown in figure 9*a*. The fineness of lotus fibres decreases with increase in treatment time from 0 min to 15 min, and then remains constant. The fineness of lotus fibres is closely related to the removal of impurities of lotus fibres. The decrease in fineness of lotus fibres results from the removal of non-cellulose substances on the surface of lotus fibres, and consequently single fibres are separated from fibre bundles. Raw lotus fibre prepared with sodium hydroxide is a kind of fibre bundle, which is formed by the adhesion of single fibres by pectin or other non-cellulose substances. Fibre bundles with a large diameter are separated into single fibres due to the removal of pectin. Raw lotus fibres have been almost separated into single lotus fibres when the treatment time is prolonged to 15 min. Therefore, the fineness of lotus fibres will remain constant with increase in treatment time from 15 min to 25 min.

### 3.10. Tensile strength and breaking elongation

The effects of treatment time on the tensile strength and breaking elongation of lotus fibres are illustrated in figure 9*b*. The tensile strength and breaking elongation of lotus fibres decrease with increase in treatment time from 0 min to 25 min. The lotus fibre is a kind of hollow fibre composed of superfine fibres and an external shell. The damage of the external shell of the lotus fibres has a detrimental effect on their tensile strength. Cellulose macromolecular chains in lotus fibres are mainly linked by hydrogen bonds which are easily broken when fibres are stretched. The number of hydrogen bonds between cellulose chains decreases with increase in treatment time. Therefore, the tensile strength of lotus fibres decreases with increase in treatment time. In addition, breaking elongation of lotus fibres decreases with increase in treatment time due to increase in the crystallinity of lotus fibres.

## 4. Conclusion

Lotus fibres with excellent moisture regain and whiteness are prepared with sodium hydroxide followed by hydrogen peroxide under microwave irradiation. The lotus fibre is a kind of natural hollow fibre which is composed of superfine fibres and an external shell. The cellulose component in lotus fibres, and their moisture regain and whiteness are improved after they are treated with hydrogen peroxide. The removal rate of impurities and the fineness of lotus fibres decrease with increase in treatment time. Microwave irradiation is a convenient and efficient method for the preparation of lotus fibres. This study opens up an opportunity for the use of microwaves in the preparation of lotus fibres.

## Data accessibility

Our data are deposited at the Dryad Digital Repository: http://dx.doi.org/10.5061/dryad.c520h [29].

## References

[1] Wu M, Shuai H, Cheng Q, Jiang L 2014 Bioinspired green composite lotus fibers. Angew. Chem. Int. Ed. 53, 3358–3361. (doi:10.1002/anie.201310656) 10.1002/anie.201310656 24648126

[2] Gong Y, Han GT, Zhang YM, Zhang JF, Jiang W, Pan Y 2015 Research on the degradation performance of the lotus nanofibers-alginate porous materials. Polym. Degrad. Stab. 118, 104–110. (doi:10.1016/j.polymdegradstab.2015.04.003)

[3] Liu D, Han G, Huang J, Zhang Y 2009 Composition and structure study of natural *Nelumbo nucifera* fiber. Carbohydr. Polym. 75, 39–43. (doi:10.1016/j.carbpol.2008.06.003)

[4] Bera A, Ghosh A, Mukhopadhyay A, Chattopadhyay D, Chakrabarti K 2015 Improvement of degummed ramie fiber properties upon treatment with cellulase secreting immobilized *A. larrymoorei* A1. Bioproc. Biosyst. Eng. 38, 341–351. (doi:10.1007/s00449-014-1274-6) 10.1007/s00449-014-1274-6 25194464

[5] Borchani KE, Carrot C, Jaziri M 2015 Untreated and alkali treated fibers from Alfa stem: effect of alkali treatment on structural, morphological and thermal features. Cellulose 22, 1577–1589. (doi:10.1007/s10570-015-0583-5)

[6] Lazic B, Janjic S, Rijavec T, Kostic M 2017 Effect of chemical treatments on the chemical composition and properties of flax fibers. J. Serb. Chem. Soc. 82, 83–97. (doi:10.2298/jsc160707106l)

[7] Li L, Sun J, Jia G 2012 Properties of natural cotton stalk bark fiber under alkali treating. J. Appl. Polym. Sci. 125, E534–E539. (doi:10.1002/app.36987)

[8] Zhang Y, Guo Z 2014 Micromechanics of lotus fibers. Chem. Lett. 43, 1137–1139. (doi:10.1246/cl.140345)

[9] Baig G, Carr C 2016 Surface and structural damage to PLA fibres during textile pretreatments. Fibres Text. East. Eur. 24, 52–58. (doi:10.5604/12303666.1191427)

[10] Kiralan M, Ramadan MF 2016 Volatile oxidation compounds and stability of safflower, sesame and canola cold-pressed oils as affected by thermal and microwave treatments. J. Oleo Sci. 65, 825–833. (doi:10.5650/jos.ess16075) 27725480 10.5650/jos.ess16075

[11] Nair GR, Singh A, Kurian J, Vijaya Raghavan GS 2016 Mathematical analysis of compound release during microwave assisted retting of flax stems. Biosyst. Eng. 150, 214–221. (doi:10.1016/j.biosystemseng.2016.08.009)

[12] Su J, Zhu H, Wang L, Liu X, Nie S, Xiong J 2016 Optimization of microwave-hydrogen peroxide pretreatment of cellulose. Bioresources 11, 7416–7430. (doi:10.15376/biores.11.3.7416-7430)

[13] Huang X, De Hoop CF, Li F, Xie J, Hse C-Y, Qi J, Jiang Y, Chen Y 2017 Dilute alkali and hydrogen peroxide treatment of microwave liquefied rape straw residue for the extraction of cellulose nanocrystals. J. Nanomater. 2017, 4049061 (doi:10.1155/2017/4049061)

[14] Han J, Zhou C, French AD, Han G, Wu Q 2013 Characterization of cellulose II nanoparticles regenerated from 1-butyl-3-methylimidazolium chloride. Carbohydr. Polym. 94, 773–781. (doi:10.1016/j.carbpol.2013.02.003) 23544632 10.1016/j.carbpol.2013.02.003

[15] Pan Y, Han G, Mao Z, Zhang Y, Duan H, Huang J, Qu L 2011 Structural characteristics and physical properties of lotus fibers obtained from *Nelumbo nucifera* petioles. Carbohydr. Polym. 85, 188–195. (doi:10.1016/j.carbpol.2011.02.013)

[16] Tang CL, Hu DM, Cao QQ, Yan W, Xing B 2017 Silver nanoparticles-loaded activated carbon fibers using chitosan as binding agent: preparation, mechanism, and their antibacterial activity. Appl. Surf. Sci. 394, 457–465. (doi:10.1016/j.apsusc.2016.10.095)

[17] Guo XH, Hang Y, Xie ZC, Wu C, Gao L, Liu CX 2017 Flexible and wearable 2.45 GHz CPW-fed antenna using inkjet-printing of silver nanoparticles on pet substrate. Microw. Opt. Technol. Lett. 59, 204–208. (doi:10.1002/mop.30261)

[18] Pan Y, Han G, Mao Z, Zhang Y, Huang J, Qu L 2011 The anatomy of lotus fibers found in petioles of *Nelumbo nucifera*. Aquat. Bot. 95, 167–171. (doi:10.1016/j.aquabot.2011.05.002)

[19] Padzil FN, Zakaria S, Chia CH, Jaafar SN, Kaco H, Gan S, Ng P 2015 Effect of acid hydrolysis on regenerated kenaf core membrane produced using aqueous alkaline-urea systems. Carbohydr. Polym. 124, 164–171. (doi:10.1016/j.carbpol.2015.02.013) 25839807 10.1016/j.carbpol.2015.02.013

[20] Reddy N, Yang Y 2009 Properties of natural cellulose fibers from hop stems. Carbohydr. Polym. 77, 898–902. (doi:10.1016/j.carbpol.2009.03.013)

[21] Shen M, Wang L, Long JJ 2015 Biodegumming of ramie fiber with pectinases enhanced by oxygen plasma. J. Clean. Prod. 101, 395–403. (doi:10.1016/j.jclepro.2015.03.081)

[22] Derkacheva OY 2015 Determination of cellulose fiber structure using IR reflectance spectroscopy of paper. J. Appl. Spectrosc. 81, 1037–1043. (doi:10.1007/s10812-015-0047-6)

[23] Kavitha SR, Umadevi M, Janani SR, Balakrishnan T, Ramanibai R 2014 Fluorescence quenching and photocatalytic degradation of textile dyeing waste water by silver nanoparticles. Spectrochim. Acta A Mol. Biomol. Spectrosc. 127, 115–121. (doi:10.1016/j.saa.2014.02.076) 24632164 10.1016/j.saa.2014.02.076

[24] Gierlinge N, Goswami L, Schmidt M, Burgert I, Coutand C, Rogge T, Schwanninger M 2008 In situ FT-IR microscopic study on enzymatic treatment of poplar wood cross-sections. Biomacromolecules 9, 2194–2201. (doi:10.1021/bm800300b) 18636773 10.1021/bm800300b

[25] Kandimalla R, Kalita S, Choudhury B, Devi D, Kalita D, Kalita K, Dash S, Kotoky J 2016 Fiber from ramie plant (*Boehmeria nivea*): a novel suture biomaterial. Mater. Sci. Eng. C Mater. Biol. Appl. 62, 816–822. (doi:10.1016/j.msec.2016.02.040) 26952488 10.1016/j.msec.2016.02.040

[26] Fan P, He F, Yang Y, Ao M, Ouyang J, Liu Y, Yu L 2015 In-situ microbial degumming technology with *Bacillus* sp. HG-28 for industrial production of ramie fibers. Biochem. Eng. J. 97, 50–58. (doi:10.1016/j.bej.2014.12.010)

[27] Pacaphol K, Aht-Ong D 2017 Preparation of hemp nanofibers from agricultural waste by mechanical defibrillation in water. J. Clean. Prod. 142, 1283–1295. (doi:10.1016/j.jclepro.2016.09.008)

[28] Zhang TH, Guo M, Cheng L, Li XL 2015 Investigations on the structure and properties of palm leaf sheath fiber. Cellulose 22, 1039–1051. (doi:10.1007/s10570-015-0570-x)

[29] Cheng C, Guo R, Lan J, Jiang S. 2017 Data from: Extraction of lotus fibres from lotus stems under microwave irradiation Dryad Digital Repository. (http://dx.doi.org/10.5061/dryad.c520h) 10.1098/rsos.170747 PMC5627114 28989774

